# Multipathway Quantitative Assessment of Exposure to Fecal Contamination for Young Children in Low-Income Urban Environments in Accra, Ghana: The SaniPath Analytical Approach

**DOI:** 10.4269/ajtmh.16-0408

**Published:** 2017-08-21

**Authors:** Yuke Wang, Christine L. Moe, Clair Null, Suraja J. Raj, Kelly K. Baker, Katharine A. Robb, Habib Yakubu, Joseph A. Ampofo, Nii Wellington, Matthew C. Freeman, George Armah, Heather E. Reese, Dorothy Peprah, Peter F. M. Teunis

**Affiliations:** 1 Center of Global Safe Water, Sanitation, and Hygiene, Hubert Department of Global Health, Rollins School of Public Health, Emory University, Atlanta, Georgia;; 2 Water Research Institute (WRI), Council for Scientific and Industrial Research (CSIR), Accra, Ghana;; 3 Training Research and Networking for Development (TREND Group), Accra, Ghana;; 4 Noguchi Memorial Institute for Medical Research, College of Health Sciences, University of Ghana - Legon, Accra, Ghana;; 5 Centre of Zoonoses and Environmental Microbiology, Centre for Infectious Disease Control, RIVM, Bilthoven, The Netherlands

## Abstract

Lack of adequate sanitation results in fecal contamination of the environment and poses a risk of disease transmission via multiple exposure pathways. To better understand how eight different sources contribute to overall exposure to fecal contamination, we quantified exposure through multiple pathways for children under 5 years old in four high-density, low-income, urban neighborhoods in Accra, Ghana. We collected more than 500 hours of structured observation of behaviors of 156 children, 800 household surveys, and 1,855 environmental samples. Data were analyzed using Bayesian models, estimating the environmental and behavioral factors associated with exposure to fecal contamination. These estimates were applied in exposure models simulating sequences of behaviors and transfers of fecal indicators. This approach allows us to identify the contribution of any sources of fecal contamination in the environment to child exposure and use dynamic fecal microbe transfer networks to track fecal indicators from the environment to oral ingestion. The contributions of different sources to exposure were categorized into four types (high/low by dose and frequency), as a basis for ranking pathways by the potential to reduce exposure. Although we observed variation in estimated exposure (10^8^–10^16^ CFU/day for *Escherichia coli*) between different age groups and neighborhoods, the greatest contribution was consistently from food (contributing > 99.9% to total exposure). Hands played a pivotal role in fecal microbe transfer, linking environmental sources to oral ingestion. The fecal microbe transfer network constructed here provides a systematic approach to study the complex interaction between contaminated environment and human behavior on exposure to fecal contamination.

## INTRODUCTION

Wherever humans live, fecal microbes are present in their environment. As small children start to explore, their behavior brings them into contact (touching, mouthing) with environmental surfaces, animate or inanimate, often more intensively than their parents.^1,2^ Particularly in urban settings with high population density and inadequate sanitation, children may be exposed to high levels of fecal contamination and the associated enteric pathogens.^3,4^

Practical efforts to reduce childhood morbidity and mortality due to enteric pathogens concentrate on improving water and sanitation infrastructure or promoting hygienic behaviors. Most interventions (except vaccinations) are aimed at decreasing the health burden by reducing exposure. The impacts of such interventions are typically measured in epidemiological studies, comparing disease outcomes in an intervention population with a control population,^5^ for instance, a randomized controlled trial. If the intervention is effective, it should lead to a reduction in disease.^6^

However, even if an intervention does reduce exposure to fecal pathogens, it is quite possible that there will be no observable concomitant reduction in diarrheal disease. As shown by Briscoe, in a highly contaminated setting, blocking a single pathway of fecal-oral exposure (or even some combination of different pathways) may still leave other, sometimes unknown, exposure pathways unchanged.^7^ In this study, the pathway was defined as any link between a specific *source* of microbes and oral ingestion (a *sink*). When postintervention exposure through these other pathways is high enough, there may be no measurable impact on population health.^7^ Therefore, the absence of an observed impact on illness does not imply that exposure must be unchanged—any intervention that blocks a specific exposure pathway is expected to reduce exposure, just not always to an extent that reduces the overall dose (i.e., the sum of contributions from all pathways) to levels that reduce the risk of acute enteric illness or other adverse health effects associated with fecal contamination (e.g., environmental enteropathy).^8–10^ It is critical to know how much each pathway contributes to overall exposure, and which pathways may be expected to have the greatest contribution. Such information strengthens the basis for designing intervention strategies.

It is for this reason, the SaniPath study was designed as an exposure assessment to complement the epidemiological approach—instead of measuring the impact of sanitation intervention on illness endpoints, we aim to quantify exposure.^8^ As exposure is a covert variable (it cannot be directly measured in an observational population study), a bottom-up approach must be used, as developed for microbial risk assessment.^11,12^ Concentrations of fecal microbes in the environment, exposure behaviors (frequency, duration, and order), and the associated intakes (amount ingested) must be quantified for each exposure pathway. Then, exposure estimates (numbers of fecal microbes ingested daily) may be calculated, including their variation among individual children, stratified by age, neighborhood, or other classifiers.

**Figure 1.**
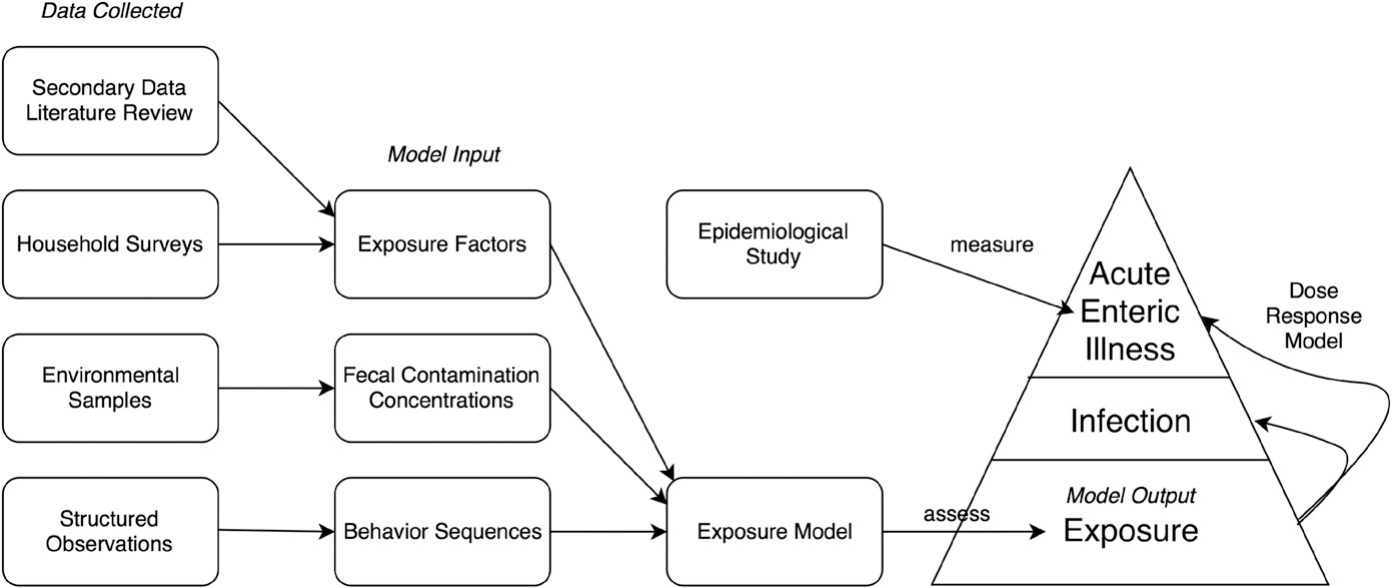
Conceptual diagram about exposure assessment framework (data collected, model input, exposure model, model output) and its relationship with epidemiological studies.

As illness is conditional on infection, and infection is conditional on exposure,^13^ the probability of exposure is greater than the probability of illness, ignoring immunity. Epidemiological estimates of illness incidence suffer from underascertainment and under-reporting.^14^ Thus, exposure risk and observed incidence of illnesses define upper and lower bounds, respectively, of the true illness rate, and complement each other (Figure 1).

The objectives of this study were to provide a systematic method for assessing the magnitudes of fecal exposure pathways and to compare multiple exposure pathways by microbial risk assessment. The exposure model described here may aid in designing water, sanitation, and hygiene intervention strategies to improve their effectiveness in reducing exposure. The model can be easily adjusted to simulate an (infrastructure or behavioral) intervention to allow exploration of the impact of those interventions on exposure.

## METHODS

The conceptual framework of this study, including the data collected, model input, exposure model, and model output, is summarized in Figure 1. This figure also shows the surveillance pyramid illustrating decreasing fractions of the population, going from exposure via infection (including asymptomatic) to observed illnesses.

### Data collected and model input.

Structured observations of behavior, environmental samples of media that children touched or ingested, household surveys about demographic information and data on water usage, sanitation, and hygiene were collected for children under 5 years living in households in four neighborhoods with a range of characteristics in the city of Accra, Ghana (January 2011 to December 2012). The study was reviewed and approved by the Institutional Review Board at Noguchi Memorial Institute for Medical Research, University of Ghana (Accra) and Emory University (Atlanta, GA).

More than 500 hours of structured observations were collected for 156 children in 23–35 households in each of the four study neighborhoods, resulting in records consisting of a sequence of activities, where these activities occurred (compartments), and durations of these activities. Activities recorded were playing/sitting, sleeping, handwashing, bathing, defecating, and eating; compartments were dirt floor, improved (concrete) floor, off-ground, stagnant water/trash area, and open drain. Definitions of these activities and compartments and primary results of behavior analysis including descriptive statistics have been previously described by Teunis et al.^15^

The rates with which children changed from one behavior to the next, were estimated in a competing hazards model. With the estimated transition rates, a simulation model was built that allowed the generation of behavior sequences—a hypothetical “day in the life” of a child, as a model for behavior.

A total of 1,855 environmental samples were collected during the study period, including liquid samples (tap water, household stored water, drain water, ocean water, flood water, irrigation water), solid samples (soil, sand, sediment, vendor food, raw produce), and surface swabs (concrete floor, off-ground surface, public latrine surface) to assess fecal indicator bacteria and virus concentrations. Additional details of study design, sampling methods, and primary results are provided by Robb et al.^8^

Concentrations of enteric pathogens vary by location and time, and the same is true for fecal indicator organisms (*E. coli* were used in this study), as a proxy for fecal contamination. For instance, fecal contamination on surfaces may vary by neighborhood—the concentrations may differ because of variation in behavior of the residents, whereas the concentrations on various types of surfaces within any household may vary because these surfaces are different materials (soil floors versus concrete floor) and used differently (floors versus table tops). All microbial concentration parameters were estimated using a hierarchical Bayesian model implemented in JAGS/rjags, using noninformative priors where possible.

Information from 800 household surveys and literature reviews, including some secondary data, was used to create exposure factors about breastfeeding, hands touching surfaces, hand mouthing, handwashing, children’s diet, and drinking water consumption.

### Exposure model.

The purpose of exposure assessment was to compare exposure pathways by estimating numbers of fecal microbes ingested by children from various sources in the environment. Exposure, the end point of the model, was defined as oral ingestion of any number of fecal microbes by a child during a certain time period (usually 1 day). Oral ingestion may occur through mouthing of a contaminated hand, or eating or drinking. Children were divided into three age groups (0–1 year old, 1–2 years old, and 2–5 years old) for this analysis because of observed differences in mobility (crawling, walking) and intake patterns of food and drink.^15^

Over a period of time, exposure depended not only on all the behaviors (characterized by type, duration) that occurred during that period, but also on the order in which they occurred. Such information was available from the behavioral analysis.^15^ There are sources of fecal contamination (drain, soil, surfaces, food, and water) and sinks (mouth, handwashing, and bathing). They are connected by multiple links resulting in a fecal microbe transfer network. Exposure through any activities that involve contact with the environment depends on the contamination at the time and location (compartment) where the contact happened. Therefore, the model of fecal microbe transfer was organized by types of actions (modules) describing the fecal-oral pathways:

1. Hand contact with fomites (hand.fomites): dirt floor/concrete floor/off-ground surfaces (soil, swabs).
2. Hand contact with open drains (hand.drain).
3. Accidental contact with child’s (own) feces associated with defecation (hand.defecation).
4. Hand mouthing (hand.mouthing).
5. Handwashing (hand.washing) and bathing (hand.bathing).
6. Eating (including breast milk, raw produce, prepared ready-to-eat food, bought food).
7. Drinking tap water or sachet water (expos.dw).

**Figure 2.**
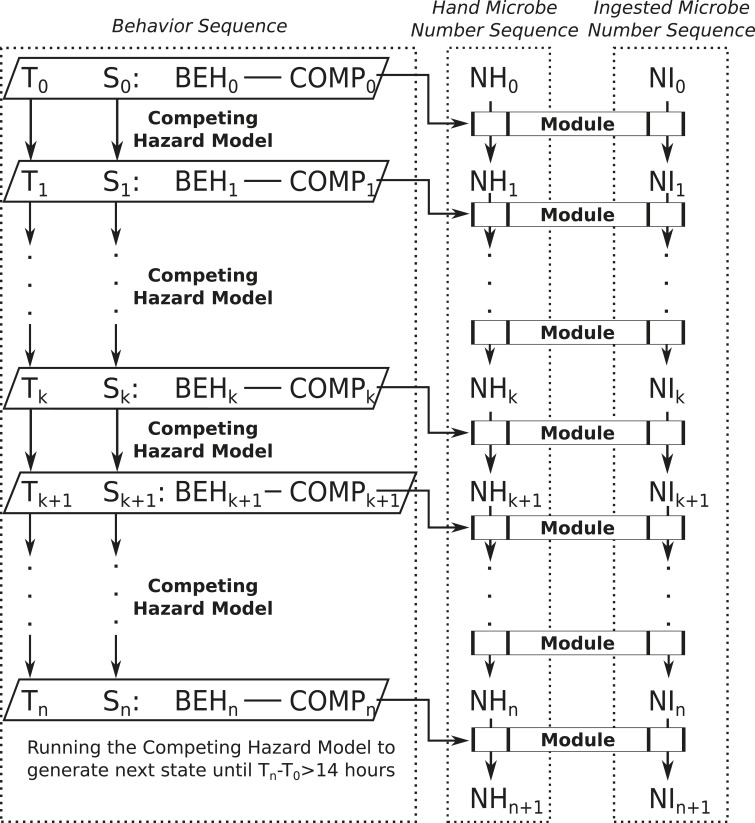
Exposure model structure. Time is denoted as “T.” State (S) is a combination of behavior (BEH) and compartment (COMP). Competing Hazard model will generate the next time (T) and state (S) until T_n_ − T_0_ > 14 hours. Behavior sequence, defined by Teunis et al.,^15^ consists of a sequence of T and a sequence of S. Microbe number on hands (NH) and microbe number ingested (NI) will also be sequences generated by exposure modules selected based on current duration (difference in time) and state.

For example, when a child is playing/sitting on a dirt floor, hand contact with fomites (soil) and hand mouthing modules are used to model occasional contact with soil and mouthing (inserting his/her hands into the mouth). To calculate the numbers of fecal microbes transferred in any particular module, functions were created to model microbial attachment and detachment of microbes from any source, including food/drinking water. Each time a child’s hand touches a surface, an exchange of microbes occurs between the hand and the surface. With repeated touching behavior, the contamination on hands reaches a steady state within a few touching events.^16^ Simulations were used to generate complete sequences of child behavior (14 hours daytime assumed), along with fecal microbe transfers, stratified by neighborhood and child age (category). Figure 2 shows the structure of the exposure model. Simulations start with behavior sequences, generated by the competing hazards model described in Teunis et al.^15^ For any simulated behavior and compartment combination, the corresponding microbe transfer module (Table 1) generates numbers of fecal microbes, on hands or orally ingested after this behavior. Depending on the type of behavior, frequency or duration is used in the calculations. The time course of numbers of microbes on hands and numbers ingested is recorded, as are the sources of any transferred microbes. Because the simulated behavioral sequences provide complete information about the child’s activities and compartments, the simulations allow tracing the sources of ingested fecal microbes throughout the network, and hence quantifying the contribution of any particular source of fecal contamination to exposure. In this study, 10,000 Monte Carlo samples were generated for all exposures during a typical child day (14 hours) for each age group and neighborhood. All simulations were run using statistical software R version 3.1.1.^17^

**Table 1:** Exposure modules link to behaviors and compartment combinations

Compartment	Behavior	Module
Dirt floor	Playing/Sitting	hand.fomites
		hand.mouthing
Concrete floor	Playing/Sitting	hand.fomites
		hand.mouthing
Off-ground	Playing/Sitting	hand.fomites
		hand.mouthing
Stagnant water and trash area/drain	Playing/Sitting	hand.drain
–	Sleeping	hand.mouthing
–	Handwashing	hand.washing
–	Bathing	hand.bathing
Dirt floor	Defecating	hand.defecation
		hand.fomites
Concrete floor	Defecating	hand.defecation
		hand.fomites
Off-ground	Defecating	hand.defecation
		hand.fomites
Stagnant water and trash area/drain	Defecating	hand.defecation
		hand.drain
–	Eating produce	eating
–	Eating prepared/bought food	eating
–	Drinking tap water	expos.dw
–	Drinking sachet water	expos.dw

Each module specifies and quantifies certain microbe transfers between sources, vehicles, and sinks in the network structure. “–” means “not applicable.”

## RESULTS

### Exposure by source.

**Figure 3.**
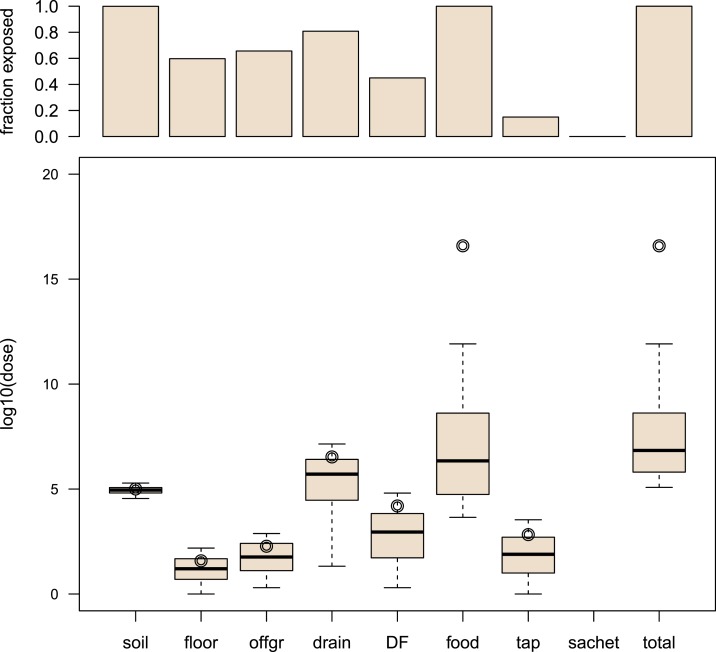
Exposure to fecal contamination from different sources for children of 2–5 years in Bukom. Top graph: bar chart of the fraction exposed (i.e., percent of child days exposed to any number of *Escherichia coli* among 10,000 simulated days). Bottom graph: boxplots of log10 dose of *E. coli* (unit of dose is CFU/day). Note that the “whiskers” show 5th and 95th percentiles instead of adjacent values. The upper/lower adjacent value is the value of the largest/smallest observation that is less/greater than or equal to the upper/lower quartile plus/minus 1.5 the length of the interquartile range. No outliers are shown in the boxplots, and the circle represents the log10 of the arithmetic mean of nonzero doses. offgr = “off-ground surfaces,” DF = “direct contact with own feces,” tap = “tap water,” sachet = “sachet water.” This figure appears in color at www.ajtmh.org.

Exposure of age group 2–5 years in Bukom, one of the four study neighborhoods in Accra, Ghana, is selected as an example to illustrate model output by source. Exposure from different sources was characterized by fraction exposed (i.e., fraction with a nonzero dose), distribution of log10 dose, and (arithmetic) mean dose.^18^ Figure 3 shows boxplots of log10 dose of *E. coli* (for exposed children) and a bar chart of fractions exposed (i.e., percent of child days exposed to any number of *E. coli* among 10,000 simulated days) for different sources of fecal contamination, for children 2–5 years old in Bukom. Both two outputs are shown because for any single source, there may be a considerable proportion of zeros, indicating that the child was not exposed to that source. In most simulations (all age groups and all neighborhoods), the food source tended to dominate overall exposure to fecal microbes. Exposure from food depended on choice of food, consumption of food, food contamination level, and food handling behavior. In exposure from drains, the dose was high, but the fraction exposed was moderate. For “direct contact with child’s own feces” (DF), the fraction exposed was very low, but if exposure occurred, the dose was high. The fractions exposed to *E. coli* in the tap water and sachet water were very small because of low contamination in those drinking water sources. Because of frequent contacts, the total doses of *E. coli* from soil, floor, and off-ground sources showed little variation, but the fractions exposed to those sources were high. Pathways that involved rare occurrences of high-dose contact events (e.g., Drain, DF) had much larger variation in exposure. Based on the exposure estimates (log10 dose and fraction exposed), we were able to classify pathways into four categories by dose (high, low) and frequency (high, low) (Table 2).

**Table 2:** Categorization of exposure pathways (designated by source) by dose and frequency

		Dose	
		High	Low
Frequency	High	Food	Soil
			Floor
			Off-ground
	Low	Drain	DF
			Tap water
			Sachet water

DF = direct contact with own feces. Frequency shows how frequent (1/days) children ingest *Escherichia coli* from sources.

### Exposure by neighborhood and age group.

**Figure 4.**
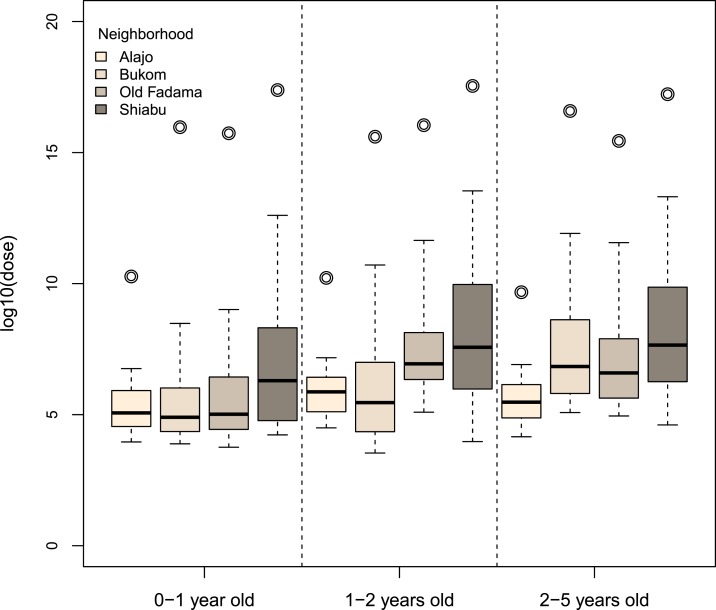
Total exposure to fecal contamination by neighborhood and age group. Boxplots of log10 dose of *Escherichia coli* (unit of dose is CFU/day). Note that the “whiskers” show 5th and 95th percentiles. No outliers are shown in the boxplots, and the circle represents the log10 of the arithmetic mean of nonzero doses. This figure appears in color at www.ajtmh.org.

The four study neighborhoods had different characteristics, with respect to population density, socioeconomic status (SES), and sanitation coverage.^19^ Figure 4 shows the overall exposure for different age groups and neighborhoods. Because the combined exposure from all pathways was never zero, the figure only includes log10 dose information. Among the four neighborhoods, young children in Alajo had the lowest total exposure, whereas young children in Shiabu had the highest. Even when living in the same environment, predicted exposures varied for children in different age groups as a result of differences in behavior, intake value (volume, weight, surface area), and choice of food. Children of age 0–1 year had lower total exposure compared with the other two age groups (1–2 years old, 2–5 years old). The detailed exposure by source, neighborhood, and age group are shown in additional graphs (Supplemental Figures A1–A7) in the Supplemental Appendix.

### Fecal microbe transfer networks.

**Figure 5.**
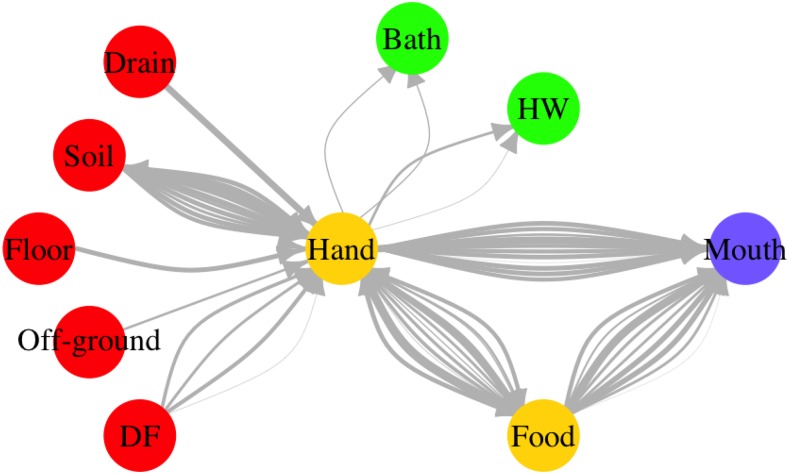
Fecal microbe transfer network for a typical child day (2–5 years old, Bukom). DF = “direct contact with own feces,” HW = “handwashing.” The weights of edges are proportional to the log10 number of microbes transferred. The color of nodes represents their role in the network. Red: sources; yellow: vehicles (can be source and sink); green: sinks (remove contamination); purple: ingestion.

**Figure 6.**
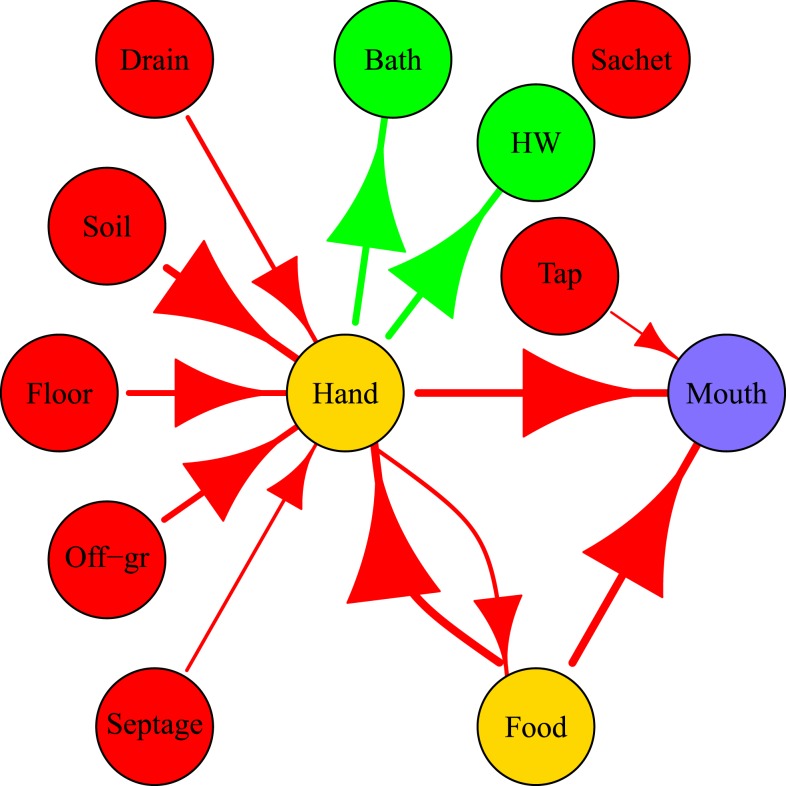
Fecal microbes transfer networks averaged over 10,000 simulated child days for children of age 2–5 years in Bukom. DF = “direct contact with own feces,” HW = “handwashing,” tap = “tap water,” sachet = “sachet water.” The size of arrows and edges are proportional to the log10 of the average numbers of fecal microbes transferred (for 10,000 simulated child days). The color of nodes represents their role in the network. Red: sources; yellow: vehicles (can be source and sink); green: sinks (remove contamination); purple: ingestion.

In the simulated exposures, specific behavioral sequences with associated fecal microbe transfers were completely known. For that reason, it was possible to keep track of fecal microbes transferred between the environment and hands and of fecal microbes ingested by the child. An example of a fecal microbe transfer network for a typical day of a 2–5-year old child in Bukom is shown in Figure 5. The widths (weights) of edges in the figure are proportional to the log10 number of microbes transferred. Variability in the weights of the edges between two nodes represents variation in number of microbes transferred through the same behavior for the same child on the same day. For example, a child can “play/sit” on the concrete floor twice on the same day, but the duration of the behavior and the concentrations of fecal microbes on the concrete floor can vary, resulting in different numbers of microbes transferred from floor to hands (Figure 5). The mouth node had no outgoing edges, as here fecal pathogens could only be ingested. Figure 5 clearly illustrates how hands played a central and crucial role in fecal microbe transfer by connecting *sources* of fecal microbes (drain, soil, floor, off-ground surface), with *sinks* (bath, handwashing, mouth), where microbes flow out of the network, and even mediating in food intake. Figure 5 shows microbe flows in individual contact events, but network representations are also useful for summarizing fecal microbe transfer for a simulated population (10,000 child days) as shown in Figure 6. Two major direct paths for human intake of fecal microbes were eating food and mouthing of hands. Intake of fecal microbes from environmental compartments (soil, floor, off-ground, drain and DF) and surfaces of foods involve hand contacts: transfer to hands and then either mouthing of the contaminated hand, or transferring fecal microbes to the ingested food stuffs. Fecal microbes on hands could be detached by hygiene behaviors (handwashing, bathing), or simply by touching less contaminated surfaces.

### Key findings.

The key findings of this study were:

1. The greatest contribution of exposure to fecal contamination was from food.
2. Children in Alajo, the highest SES neighborhood, had the lowest total fecal exposure, whereas children in Shiabu had the highest.
3. Older children (2–5 years old) had the highest total fecal exposure.
4. Hands played a central and critical role in transferring fecal microbes from the environment to oral ingestion.

## DISCUSSION

When multiple pathways contribute to fecal-oral transmission of disease, it is not feasible to empirically test all possible interventions using epidemiological methods. Therefore, it is a challenge for decision-makers in countries with poor water, sanitation, and hygiene to identify where the greatest public health risks lie and to prioritize interventions based on the most urgent needs.^20^ Our exposure assessment provides an independent probabilistic approach, to support causal analysis of the relations between interventions and health effects^21^ and guide water, sanitation, and hygiene intervention decisions in complex environments with multiple transmission pathways. The results of this comprehensive simulation allow us to predict and compare exposures and to identify important environmental exposure pathways.

### Characteristics of exposure pathways.

Generally, the higher the fecal microbe concentration and the longer the duration spend in any environment (or the higher the frequency of exposure), the higher the exposure from a specific pathway will be. Based on the characteristics (dose and fraction exposed) of the exposure pathways, we can categorize exposure pathways into four categories (Table 2)

1. High frequency and high dose: High priority pathway. Example: exposure via all the pathways with food (specifically fresh produce) as source. Interventions reducing probability of contact with fecal contamination (e.g., using clean cutlery instead of hands for eating) and reducing environmental fecal contamination (e.g., improved food hygiene and safer agricultural practices) are both important.
2. Low frequency, but high dose: Event-like exposure (exposures due to rare contact events with highly contaminated sources). Example: direct contact with drains. Interventions must be aimed at reducing the frequency of these high-dose exposure events. For example, covering drains to reduce contact rates.
3. High frequency, but low dose: Chronic exposure. Example: contact with floor/off-ground surfaces. For such pathways, it may be difficult to reduce frequency, but possibly the dose is low enough to make these pathways a low priority for intervention.
4. Low frequency and low dose: Poses little risk and not a priority for intervention. Example: drinking sachet water.

### Variation in exposure by neighborhood and age category.

Variation in behavior drives the variation in exposure among children by age (Figure 4, Supplemental Figures A1–A4). As children gain more mobility as they grow, exposure from off-ground and floor sources, that are located mainly in the private domain, decrease. At the same time, exposure from soil and drains that are located mainly in the public domain, increase as children age. The choice of food, frequency and amount of food consumed, daily water consumption, and hygiene behavior are also different for children in different age groups. Exclusive breastfeeding, which occurs in the 0–1 year age category, greatly reduces exposure via the food pathways and may provide additional protective effects against enteric diseases.^22–24^ As children grow older, the consumption of breast milk decreases whereas exposure to solid food and drinking water becomes more important.^22,25^ Note that children of different age categories may have different susceptibility to fecal pathogens. Older children, who have a more developed immune system and are more likely to have acquired immunity from prior infection, may require higher levels of exposure to fecal contamination to develop enteric illness.

Exposure estimates for the same age group also varied by neighborhood (Figure 4, Supplemental Figures A5–A7). The primary factor causing this variation by neighborhood was differences in environmental contamination—the more fecal microbes that were present where children lived, the higher their exposure. Some neighborhood characteristics, like SES, population density, geographic factors, education, and access to sanitation, have been reported to be associated with exposure to fecal contamination.^26,27^ In this study, children in Alajo, the highest SES neighborhood, had the lowest total exposure to fecal contamination, whereas Shiabu, a mixed (formal and unplanned) settlement with a highly contaminated river, had the highest total exposure (neighborhood characteristics described by Peprah et al.^19^ and Robb et al.^8^). Less latrine access and more open defecation, in neighborhoods such as Old Fadama, were likely to lead to higher fecal exposure via soil, floor, off-ground, and direct contact with child’s own feces.

### Fecal microbes transfer network.

One of the innovative aspects of this study is the use of a network approach to describe fecal microbes transfer. To better design an intervention that effectively reduces the numbers of fecal microbes ingested, not only the origin of these fecal microbes must be known, but also how they are propagated within the exposure network, via the hands to the mouth of the child.^2^

**Figure 7.**
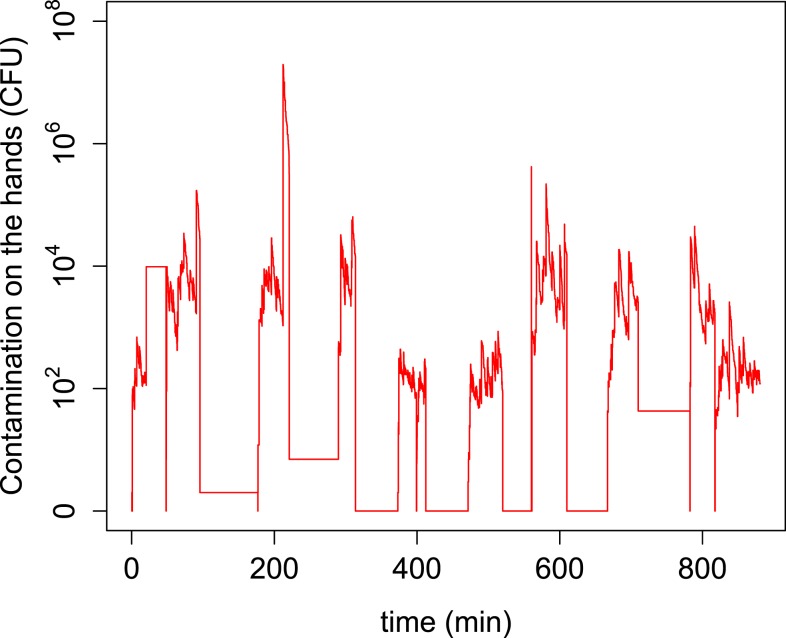
Numbers of fecal microbes on the hands for a simulated child day. This figure appears in color at www.ajtmh.org.

Pickering et al.^28^ reported a significant association between fecal contamination (titers) on hands and prevalence of diarrheal disease symptoms. Figures 5 and 6 clearly illustrate that hands occupy a central position in the transfer network as they are the hub connecting all fecal exposure pathways. Fecal microbes on any surfaces are transferred to the mouth through hands. However, large numbers of microbes transferred from the environment to hands do not necessarily imply high exposure. For example, although large numbers of microbes are transferred from soil, floor, and off-ground surfaces to hands (Figure 6), only a small portion of these microbes are ultimately ingested (Figure 3). This is a consequence of frequent and rapid temporal changes in hand contamination. Figure 7 shows a large variation of fecal contamination on hands for an individual child in the model. Data from the SaniPath study also confirms there is large variation in fecal contamination of handrinse samples (*E. coli* concentration ranged from 2.25 to 1.55 × 10^5^ CFU/pair of hands, *N* = 287, result not shown). For that reason, hand rinse sampling at any single time point becomes unpredictable and use of a small number of hand rinse data to estimate exposure to fecal microbes may be problematic. Also, because hands function as a vehicle for ingestion, there is a “memory effect” for hand contamination, which means hand contamination is determined by prior behaviors. Therefore, exposure associated with a certain behavior will be influenced by the increase or reduction of hand contamination from the preceding behaviors.

In study neighborhoods at Accra, the outer surface of raw produce can be highly contaminated. During food preparation for consumption (washing, cooking, peeling), it is the hands which come into contact with the food surfaces and the ready-to-eat food parts. During handling, fecal microbes are transferred to and from the hands and the produce surfaces to the ingested portion of the produce. Based on our findings of fecal microbes transfer networks, interventions that focus on food preparation, food handling behavior, and hygiene behaviors, such as hand washing and bathing, may be efficient at lowering the total exposure to fecal contamination for children in this study.

### Critical control points of exposure.

To achieve a reduction in exposure to fecal contamination, it is important to identify the factors that cause an important contribution to exposure. In food safety, any factors that allow control of risk are called “control points,” and when the effect of a factor is large enough to reduce the risk substantially, it is called a “critical control point.” A quantitative risk model provides a basis for Hazard Analysis by Critical Control Points (HACCP).^29,30^ To identify critical control points for reducing exposure, the influence of each factor in the model can be studied. The same approach may be used for control of the risk in fecal waste management.^31^

From the results of the current study, we can identify a few control points for fecal exposure. First, the concentrations of fecal microbes in any environment are the primary contributor to the variation in exposure among neighborhoods. Second, frequency, duration, and order of behaviors diversify children’s exposure to fecal contamination in any environment. For example, the same behavior, such as handwashing, will more likely reduce exposure to fecal contamination if it occurs *before eating* compared with if it is done *after eating*. Lastly, because the pathways from food sources dominate total exposure to fecal contamination, variables that determine food choice and food intake are important for exposure. For instance, ready-to-eat salads may be highly contaminated and consumers may ingest large numbers of microbes when eating salad. Therefore, if children eat more cooked foods, as opposed to street vended salads, exposure to fecal contamination may be lower.

The goal of this study is to present the exposure model, and summarize its application to the study neighborhoods in Accra. Detailed study of the sensitivity of these outcomes to any of the hundreds of variables, coefficients and parameters will be described separately, in a subsequent paper. In addition, factors in the model can be adjusted to simulate an (infrastructure or behavioral) intervention, which enables the model to explore the impact of specific interventions on exposure. For example, we can predict the effect of handwashing education on exposure by changing some variables in the model.

### Reducing exposure to fecal contamination.

In this study, we focused on quantifying exposure to fecal contamination which determines the probability that a child will become infected with an enteric pathogen. Infection, in turn, is a necessary (but not sufficient) condition to develop an adverse health outcome, such as gastroenteritis, environmental enteropathy, and/or stunting.^9,10,32^ To reduce the infection pressure from enteric diseases, we must reduce exposure to fecal contamination. Any reduction in exposure for the dominant pathways (food) can lead to a potentially substantial reduction of the total exposure. Note that, aside from direct changes to the dominant pathways, other changes may indirectly influence the dominant pathways to reduce exposure. For example, given that the food pathways dominated exposure (Figure 3), changes to drinking water quality are not likely to reduce overall exposure. However, it is likely that improvements to water quantity and quality do not merely reduce the exposure to fecal contamination from drinking water, which would be inappreciable to total exposure in Accra. Improved water supplies may also facilitate better hygiene by enabling more frequent hygiene behavior, safer food preparation, and a cleaner residential environment,^33^ which would act together to decrease fecal exposure. Previous intervention studies that did not observe a reduction in disease may have tested interventions that did not sufficiently lower the fecal exposure from the dominant pathways.^7^

Average fecal exposure via high dose and low frequency pathways (e.g., exposure to drains) often may be low, but at the same time, there is a small probability of a peak event—short-term exposure to a very high dose. Therefore, interventions that prevent occurrence of such outlier doses are expected to have the greatest predicted health impact. In the study by Moe et al.,^34^ there was no difference in diarrhea prevalence between groups of children with low/moderate exposure (< 1 CFU/100 mL intake, 1–10 CFU/100 mL intake, 10–100 CFU/100 mL intake, and 100–1,000 CFU/100 mL intake) to fecal contamination (*E. coli*) in drinking water. Only when exposure was beyond a certain level (> 1,000 CFU/100 mL), significantly higher diarrhea prevalence was observed.

Even significant decreases in total exposure may not necessarily result in a reduction of enteric disease. To translate exposure into health impact, we can consider the dose–response relation for infection and illness. Many different models have been used,^35–38^ but all these models agree that the probability of infection increases with increasing dose, and at high doses, the probability of infection approaches 1 (certain infection). The probability of illness (acute gastroenteritis) often increases with dose, but may saturate at some level below 1, so that even at very high doses, not everyone becomes ill because of innate or acquired immunity.^13,39^ Therefore, when exposure is high, the probability of illness may be saturated, meaning that a decrease in exposure may not result in a notable decrease in illness probability. Only a major reduction resulting in low exposure levels may result in a decrease in observable illness rates.^7^

### Public health implications for Accra.

The major finding of our exposure assessment in Accra, Ghana, is that the pathways associated with food sources are dominant for children under 5 years old, regardless of neighborhood and age group. Drechsel and Keraita^40^ observed that there are multiple opportunities for food to become contaminated “from the farm to fork” in Accra. First, wastewater irrigation and manure application may introduce a large amount of fecal contamination to raw produce items at urban farms. During harvesting, those raw produce items are collected with dirty hands and rinsed with water potentially contaminated with sewage. Second, raw produce is handled with dirty hands, it is placed on dirty surfaces, and collects a cover of fecal-contaminated dust when displayed at markets. Furthermore, dirty hands and surfaces may contact the food during food handling and preparation in the household kitchens/food vendors as well as during eating in a cultural context where most foods are traditionally eaten with hands. Contact between hands and food was modeled explicitly as we illustrated in the previous “fecal microbes transfer network” section.

Food-related pathways are complex, and there are multiple critical control points that could be addressed to reduce fecal contamination of food. At the farm, choice of irrigation water can determine the magnitude of fecal contamination on produce. In Accra, open drains are highly contaminated because of toilets that discharge directly to the drains^41^ and drain water is frequently used to irrigate produce.^40^ Preventing fecal contamination from entering open drains via better fecal sludge management, treatment of open drain water before use for irrigation, using drip irrigation instead of watering cans, or choosing cleaner, alternative sources of irrigation water are the measures that have been recommended to improve produce safety.^42,43^ During food preparation, hygienic food preparation practices for both food vendors and in household kitchens may reduce contamination on produce directly before consumption.^44^ The importance of handwashing before eating to reduce fecal exposure is demonstrated in this study (fecal microbes transfer network section).

### Strengths and limitations of the exposure model.

The SaniPath study collected lots of primary data on microbiology testing for various environmental samples, structure observation for children behavior, and surveys about exposure information. In contrast to previous studies,^45,46^ the present study combined quantitative models of child behavior with models of intake and contamination of environmental media to predict exposure to fecal microbes from multiple environmental pathways. The inclusion of a detailed quantitative model of child behavior is a unique strength of this exposure assessment. Another strength is the application of network approach in fecal microbe transfers, which help us identify and understand critical control points to reduce exposure to fecal contamination.

Despite thousands of environmental samples and hundreds of hours of behavior observations, many of the factors in the exposure model depended on secondary data from literature. Sometimes information was lacking in the published literature, so that assumptions had to be made. For example, breastfeeding frequency information for different age groups is from literature with appropriate assumptions. To improve the model, better data are needed to quantify hand transfer coefficients—Are microbial attachment and detachment coefficients associated with contamination levels on the surface of the hands, the material of the surface, the moisture level of the surface, and the duration of contact? Are the microbial reduction coefficients for handwashing/bathing associated with the duration of handwashing/bathing, and how does soap influence the reduction coefficients? Furthermore, additional information that could be collected by structured observations includes hand contact frequencies (with surfaces); ingestion of beverages (tap water, bottled water, soft drinks), frequency of breastfeeding; food consumption and food handling behavior; social contact behavior (child–child, caregiver–child contacts). Additional environmental data collection could include contamination of the outside of water sachets; more food samples and more food types to assess variability in food contamination.

## CONCLUSION

This exposure assessment model uses microbiological data on environmental contamination and observation data on behavior to develop a comprehensive quantitative evaluation of multiple exposure pathways to fecal contamination. The most influential exposure pathway(s) and its characteristics can be identified to help prioritize the interventions that could effectively reduce the total exposure to fecal contamination and the health risks from this exposure. Food pathways are dominant for childhood fecal exposure in Accra, Ghana. The fecal microbe transfer network demonstrates the importance of hands for multiple exposure pathways. The exposure assessment model allows us to use computer simulation to estimate the impact of infrastructure and behavioral interventions on different exposure pathways. The next steps for the SaniPath study are to predict the exposure (and potential health) impact of various public domain and private domain interventions and to estimate their cost-effectiveness.

## Supplementary Material

Supplemental Figure.
