# Supplementary material for: Multipathway Quantitative Assessment of Exposure to Fecal Contamination for Young Children in Low-Income Urban Environments in Accra, Ghana: The SaniPath Analytical Approach
Source: Am J Trop Med Hyg. 2017 Aug 21;97(4):1009–19. doi: 10.4269/ajtmh.16-0408 (PMC5637579; doi:10.4269/ajtmh.16-0408)
Supplement: Supplementary file 1 [file tpmd160408.SD1.pdf]

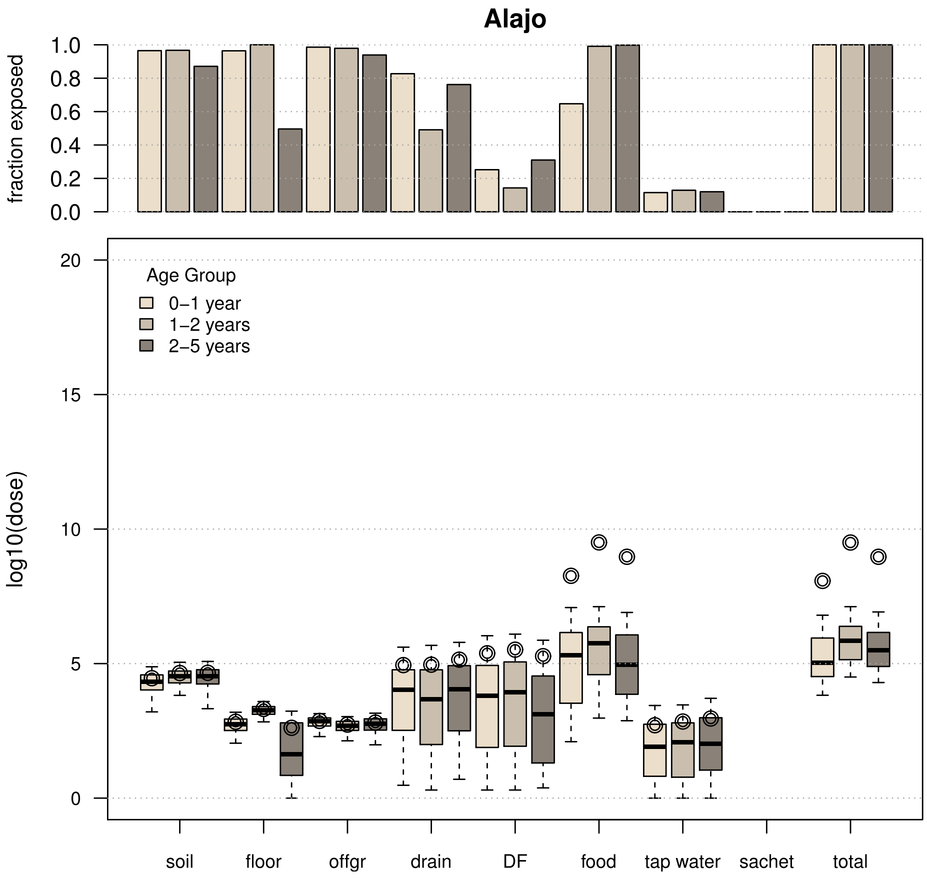

Supplemental Figure A1. Exposure from different sources for Alajo by age group.

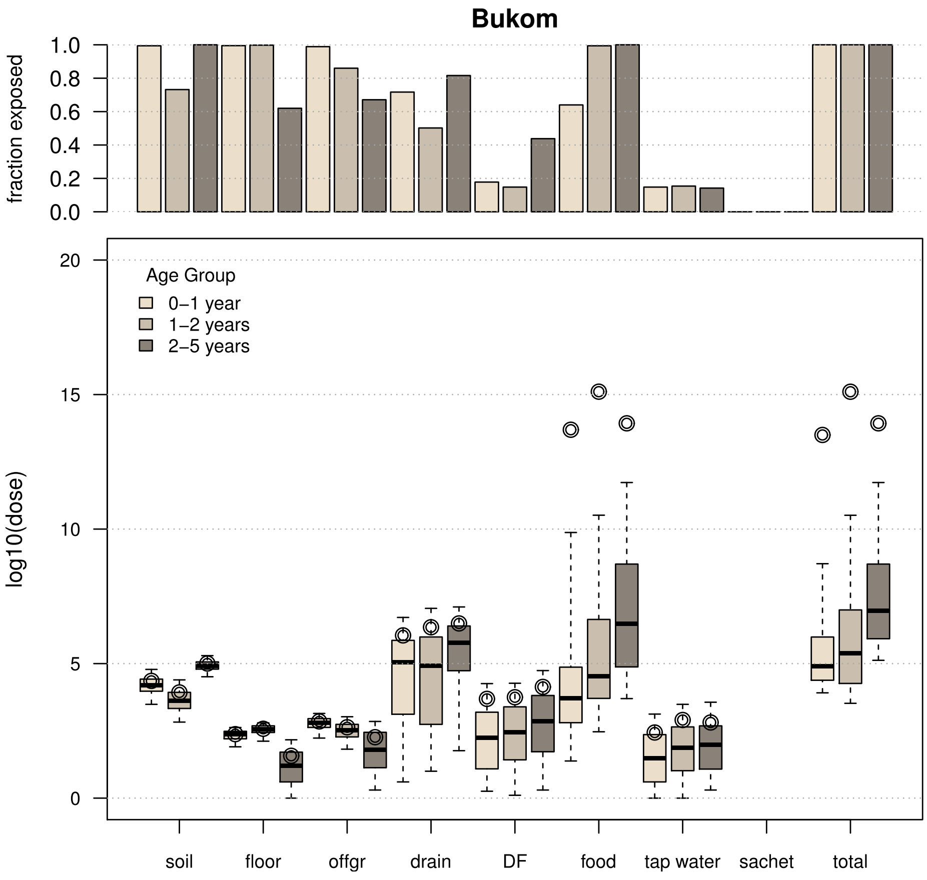

SUPPLEMENTAL FIGURE A2. Exposure from different sources for Bukom by age group.

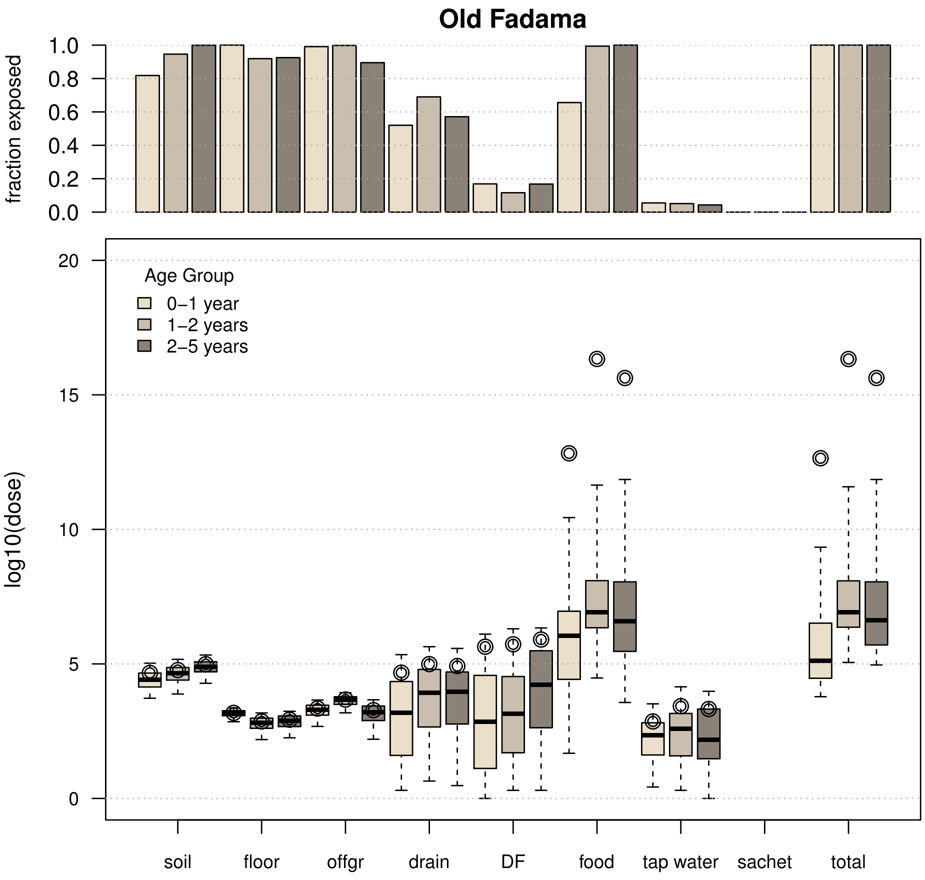

Supplemental Figure A3. Exposure from different sources for Old Fadama by age group.

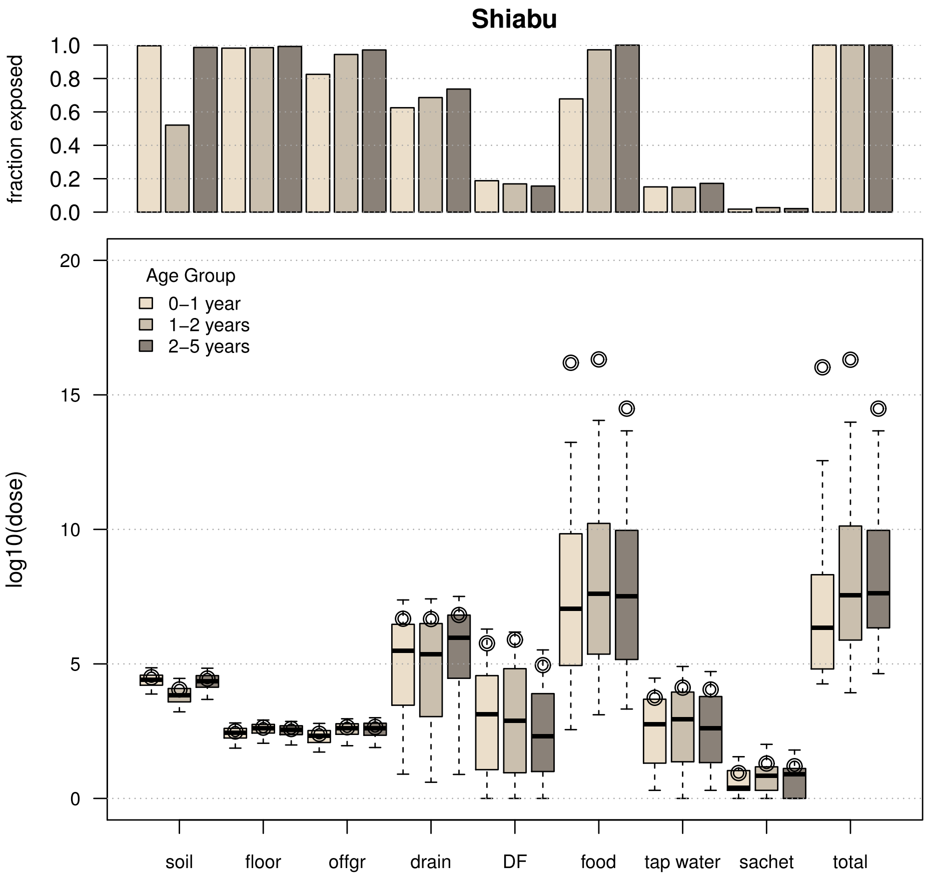

Supplemental Figure A4. Exposure from different sources for Shiabu by age group.

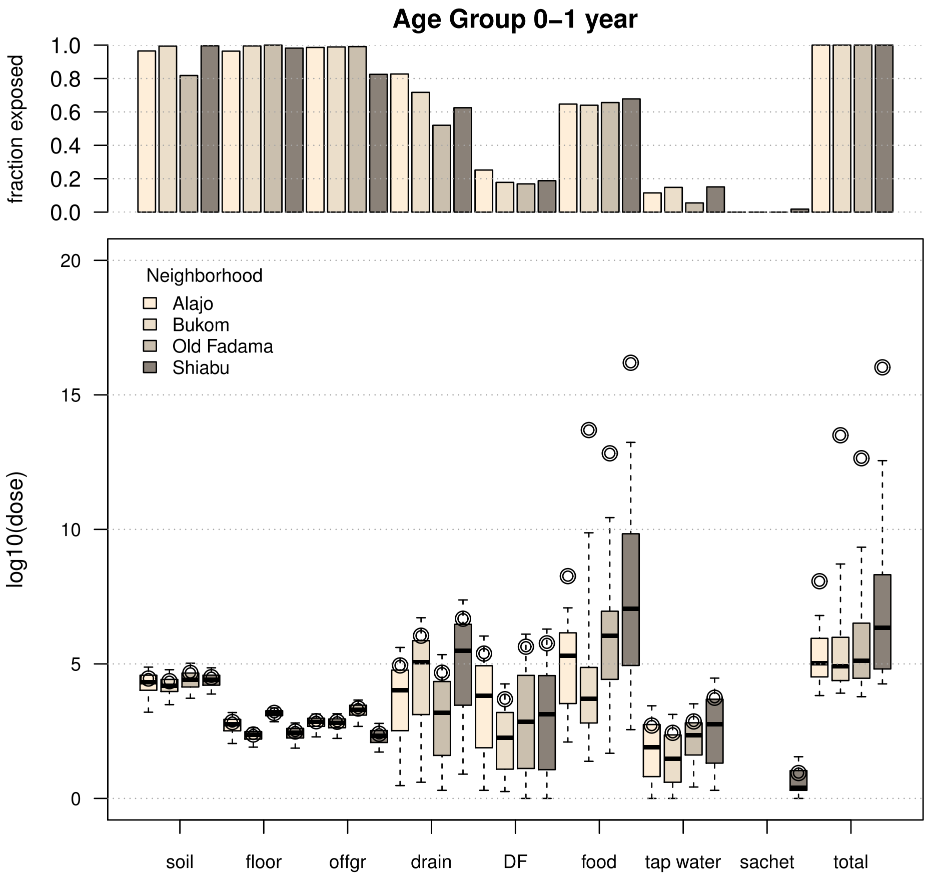

Supplemental Figure A5. Exposure from different sources for age group 0–1 year by neighborhood.

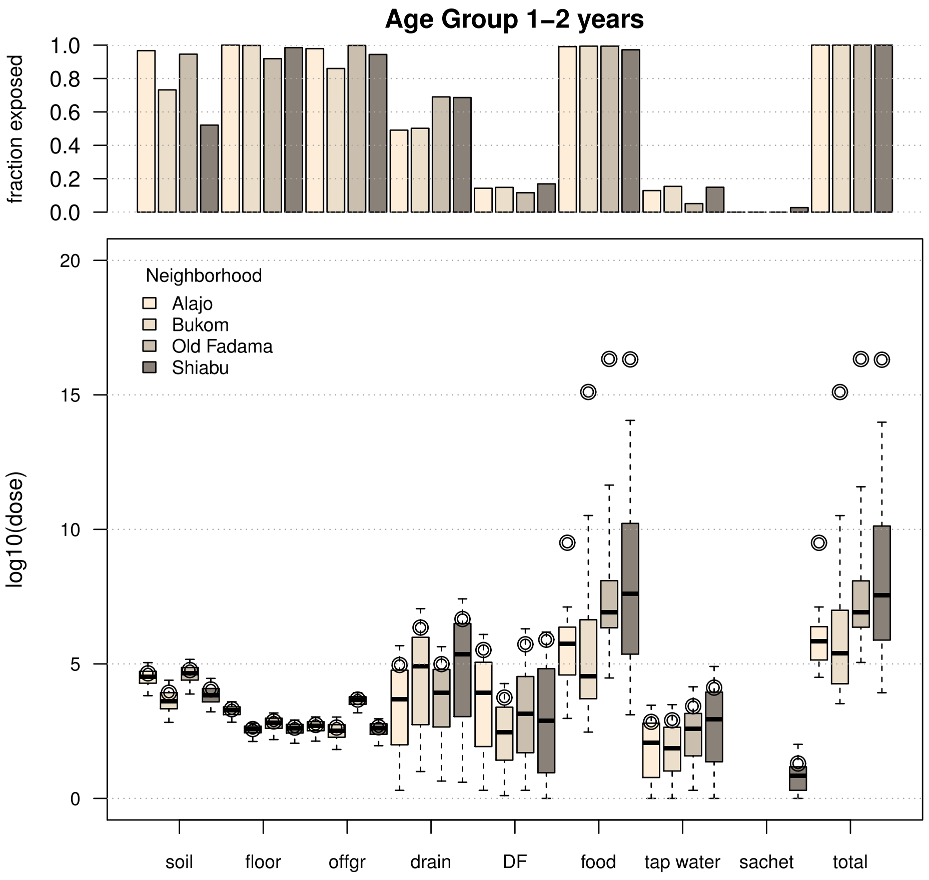

Supplemental Figure A6. Exposure from different sources for age group 1–2 years by neighborhood.

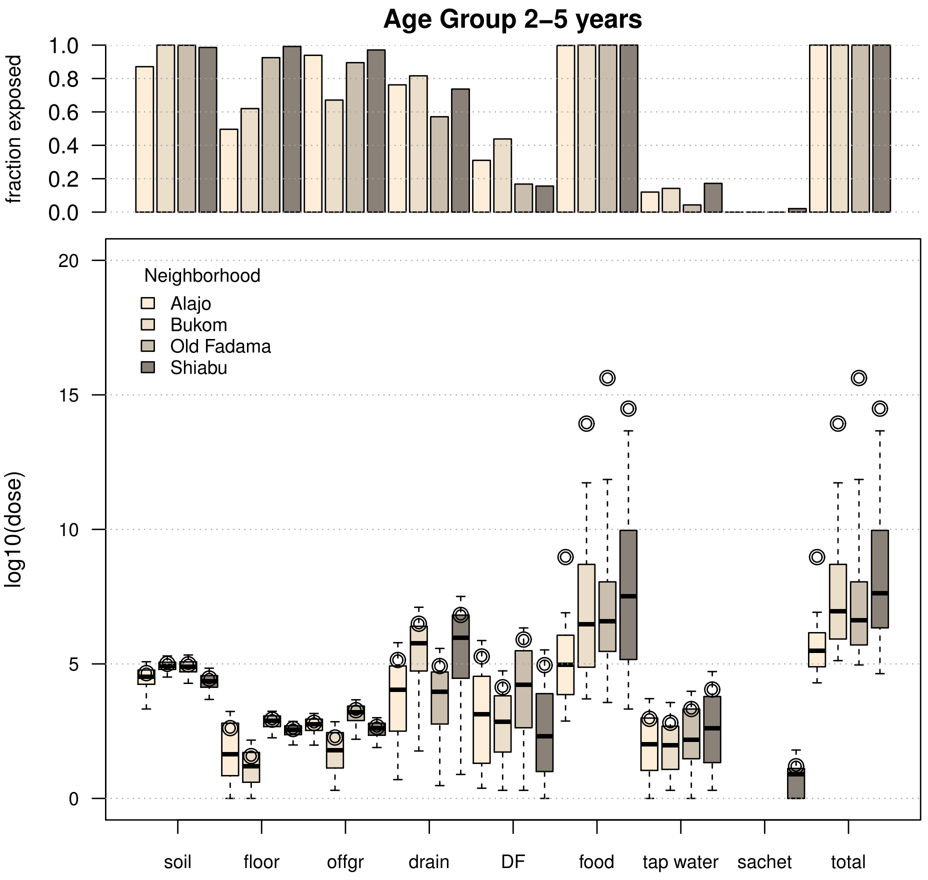

Supplemental Figure A7. Exposure from different sources for age group 2–5 years by neighborhood.
